# Jenseits von Darwin und Galton? Wissenshistorische Forschungen zu Menschenzucht und Eugenik, ca. 1600–2000

**DOI:** 10.1007/s00048-023-00361-2

**Published:** 2023-07-13

**Authors:** Pascal Germann

**Affiliations:** Institut für Medizingeschichte, Universität Bern, Bühlstraße 26, 3012 Bern, Schweiz

**Lorenz, Maren 2018:** ***Menschenzucht. Frühe Ideen und Strategien, 1500–1870. ***Göttingen: Wallstein, geb., 416 S., 24 Abb., 39,00 €, ISBN: 978-3-8353-3349‑9.

**Liggieri, Kevin 2020:** ***„Anthropotechnik“. Zur Geschichte eines umstrittenen Begriffs.*** Konstanz: Konstanz University Press, brosch., 364 S., 24 Abb., 38,00 €, ISBN: 978-3-8353-9117‑8.

**Teicher, Amir 2020:** ***Social Mendelism: Genetics and the Politics of Race in Germany, 1900–1948. ***Cambridge, MA: Cambridge University Press, geb., 280 S., 24 Abb., 29,99 £, ISBN: 978-1-10849-949‑1.

Die historische Erforschung der Eugenik verspricht Zusammenhänge zu erhellen, die über ihren eigentlichen Gegenstand hinausweisen. Erstens verkörpert die Eugenik – wie kaum eine andere Strömung – Ambivalenzen, die für die Moderne kennzeichnend sind. Sie ist integraler Bestandteil sowohl der großen Rationalitäts‑, Gesundheits- und Wohlfahrtsbestrebungen des 20. Jahrhunderts als auch seiner globalen Gewalt- und Rassismusgeschichte. Zweitens stellt die Eugenik keinen Unfall in der Entwicklung moderner Wissenschaften und der Medizin dar, sondern ist in diese eingebettet, und gerade deshalb fordert ihre Geschichte besonders nachdrücklich dazu auf, das Verhältnis von Wissenschaft und Ideologie jenseits normativer Trennungen neu zu denken. Drittens ist die Geschichte der Eugenik auf irritierende Weise unabgeschlossen. Angesichts der enorm erweiterten Möglichkeitsräume, die Reproduktions‑, Gen- und Diagnosetechnologien in den letzten fünfzig Jahren geschaffen haben, stellt sich die Frage nach einer Gegenwart und Zukunft der Eugenik mit politischer und ethischer Dringlichkeit.

Vor diesem Hintergrund ist es nicht erstaunlich, dass das große historische Interesse an der Eugenik seit mehr als drei Jahrzehnten anhält. Die Forschungsliteratur ist mittlerweile enorm angewachsen und ermöglicht heute ein differenziertes Bild. Es zeigt die globale und transnationale Dimension der Eugenik ebenso auf wie seine heterogenen lokalen Erscheinungsformen, seine Adaption an unterschiedliche ideologische, epistemische und technologische Kontexte sowie seine Verbindungen zur Geschichte von Nationen, Imperien und postkolonialen Staaten (Adams 1990; Bashford & Levine 2010; Kühl 2014; Turda & Gillette 2014; Hopwood et al. 2019). Als gemeinsamer Nenner dieser unterschiedlichen Ausprägungen der Eugenik verweist die Forschung oft auf ihre Modernität (Turda 2010). In politischer Hinsicht war die Eugenik modern, weil sie eng mit der Herausbildung von Nationalstaaten verbunden war. Zudem erwies sie sich als anschlussfähig an die großen Ideologien der Moderne. So besaß sie nicht nur eine enorme Anziehungskraft für faschistische und andere antidemokratische und nationalistische Strömungen, sondern entfaltete auch unter liberalen oder sozialistischen Vorzeichen politische Wirksamkeit (Lulay 2021; Lucassen 2010; Habermas 2002). Sexualitätshistorisch lässt sich die Eugenik als modernes Phänomen verstehen, weil sie oft mit Liberalisierungsbestrebungen einherging und zur Trennung von Sexualität und Reproduktion beitrug (Wecker et al. 2013; Bergmann 1992). Als spezifisch modern ist schließlich die enge Beziehung der Eugenik zur Wissenschaft zu nennen – mit ihrem emphatischen Bezug zu Rationalität und wissenschaftlichem Fortschritt sowie ihrem technokratischen Versprechen, soziale Probleme wissenschaftlich zu lösen. Historische Darstellungen heben dabei besonders einen Kontext hervor, der als wichtigstes Fundament der Eugenik gilt und ihre Modernität einmal mehr unterstreicht: den Darwinismus. Dementsprechend ist im *Oxford Handbook for the History of Eugenics* – einem der bedeutendsten Standardwerke zur Geschichte der Eugenik – gleich der erste Artikel dem „Darwinian Context“ gewidmet (Paul & Moore 2010). Die Geschichte der Eugenik mit dem Darwinismus beginnen zu lassen ist denn auch naheliegend. Bekanntlich war es Darwins Cousin Francis Galton (1822–1911), der im Jahr 1883 den Begriff *eugenics *prägte, um damit eine von ihm geforderte Wissenschaft zu benennen, die das Menschengeschlecht durch gezielte Zuchtmaßnahmen zu verbessern gedachte. Ebenfalls bekannt ist, wie stark Galtons Ideen von Darwins (1809–1882) *On the Origin of Species* inspiriert waren. Seit den 1860er Jahren drehten sich seine Arbeiten maßgeblich um die Frage, welche Konsequenzen aus Darwins Denken für den Menschen zu ziehen seien, und er kam zu dem Schluss, dass die menschliche Evolution planvoll gesteuert werden müsse, um einen biologischen Niedergang zu verhindern (Paul & Moore 2010: 28–34).

Die Bedeutung des Darwinismus für die Herausbildung und Entwicklung der Eugenik ist unbestritten. Der starke Fokus auf den Darwinismus und die übliche Bezeichnung Galtons als Vater der Eugenik bergen aber die Gefahr, die Geschichte der Eugenik zu sehr vom britischen Kontext her zu denken und die Vielfalt von Wissensfeldern auszublenden, welche sie mitprägten oder in historischer Verbindung zu ihr standen. Es lohnt sich deshalb, den Blick über Galton und Darwin hinaus zu weiten. Neue Zusammenhänge erhellen dementsprechend Untersuchungen zur Epoche nach 1945. Sie beleuchten Kontinuitäten und Umformungen der Eugenik in der Ära der Genetik (Löwy 2018; Cowan 2008; Burdett 2006) oder arbeiten Prozesse des „unlearning eugenics“ (Herzog 2018) im Kontext von gesellschaftlichen Auseinandersetzungen um Reproduktions- und Behindertenrechte heraus. Statt darauf zu fokussieren, was „nach“ der Eugenik kommt, ließe sich jedoch auch das in den Blick nehmen, was davor und daneben geschieht, indem die Perspektive erweitert wird auf noch wenig beachtete und weiter zurückreichende Vorstellungen der Bevölkerungsverbesserung und Menschenzucht sowie auf die Heterogenität von wissenschaftlichen Begriffen, Theorien, Diskursen und Praktiken, die Projekte der biologischen Humanoptimierung vorbereiteten, befeuerten oder mitformten. Für dieses Unterfangen ist es hilfreich, einige neuere Studien zusammenzubringen, die nicht explizit der historischen Eugenikforschung entstammen (keine der hier besprochenen Monografien trägt den Begriff Eugenik im Titel), aber relevante Aspekte der Vorgeschichte und wissenshistorischen Kontextualisierung der Eugenik beleuchten, die in dominanten Narrativen zur Eugenik noch wenig Beachtung gefunden haben.

## Menschenzucht vor Galton

Maren Lorenz’ Monografie *Menschenzucht. Frühe Ideen und Strategien, 1500–1870 *stellt einen zentralen Konsens der historischen Eugenikforschung infrage – nämlich dass die Ursprünge der Eugenik dezidiert (hoch-)modern seien. So verweist Lorenz’ Buch auf die längere Vorgeschichte der Eugenik, auf die genealogischen Verbindungen zu Vererbungs- und Bevölkerungsdiskussionen in der Frühen Neuzeit und Sattelzeit. Vorangegangene Studien haben solche Kontinuitäten schon herausgearbeitet. So argumentiert etwa John Waller (2002), Galtons Ideen seien kaum innovativ gewesen, sondern schöpften aus medizinischen Diskursen, die bereits in der ersten Hälfte des 19. Jahrhunderts vor den Gefahren der erblichen Transmission von Pathologien warnten. Lorenz’ Monografie ist aber allein schon aufgrund des umfangreichen Quellenmaterials hervorzuheben, das von philosophischen, wissenschaftlichen und medizinischen Abhandlungen über Rechtsdokumente bis zu literarischen Texten und populären Ratgebern reicht. Auch in geografischer Hinsicht spannt die Studie einen weiten Bogen: Sie behandelt Entwicklungen im Alten (Deutschen) Reich, in Frankreich, Großbritannien und den USA, wobei auch die Kolonien sowie reichs- und landesübergreifende Austauschbeziehungen in den Blick geraten. Damit zeigt sie die enorme Vielfalt von Kontexten, in denen politische Machtträger, Philosophen, Naturforscher, Ökonomen und Mediziner bereits lange Zeit vor Darwin und Galton Analogien aus der Pflanzen- und Tierzucht benutzten, um Visionen der Menschenverbesserung zu skizzieren und eine verstärkte Kontrolle des Zeugungsverhaltens zu fordern.

In ihrer Einleitung breitet Lorenz ein Panorama von Überlegungen und Projekten zur Menschenverbesserung aus, das von Sparta und Platon (ca. 428–348 v. Chr.) über die moderne Eugenik bis zur Gegenwart von Reproduktionstechnologien und pränataler Diagnostik reicht. Dabei entsteht ein düsteres Bild einer angeblich zunehmenden Verwirklichung von Optimierungsideen, das wenig Raum für Differenzierungen lässt und auf dünner Literaturgrundlage basiert. So finden sich etwa zur Geschichte der Zwangssterilisationen überzeichnete Aussagen, die in der Fußnote mit einem Aufsatz von 1967 belegt werden, was angesichts der breit vorliegenden sowie differenziert argumentierenden neueren Forschung zum Thema bedauerlich ist.

Wer jedoch lediglich die Einleitung dieser Studie liest, verpasst Grundlegendes: Auf die etwas missglückte Heranführung ans Thema folgen sechs sehr erkenntnisreiche Kapitel, die immer wieder neue und überraschende Einsichten in die enorme Heterogenität von reproduktiven Vorstellungen, Kontrollpraktiken und Züchtungsvisionen bieten. Während das erste und das sechste transnational beziehungsweise transimperial angelegt sind, widmen sich die Kapitel zwei bis fünf jeweils einem der vier behandelten Länder beziehungsweise Reiche. Der zeitliche Fokus liegt dabei auf der Zeit der Aufklärung bis zur Mitte des 19. Jahrhunderts. Historisch weiter zurück blickt das kurze erste Kapitel: Es zeigt, dass bereits utopische Schriften frühneuzeitlicher Denker Vorschläge zur qualitativen Verbesserung des Nachwuchses enthielten. Hier wie auch in späteren Kapiteln stellt sich indessen die Frage, ob der Begriff der „Menschenzucht“ nicht zu eng ist, um der Vielfalt dieser Ideen gerecht zu werden.

Wie das zweite Kapitel zum Alten Reich darlegt, zirkulierten im Zeitalter der Aufklärung vermehrt Reformvorschläge, die auf gesunden Nachwuchs und die Verhinderung von angeborenen Defekten abzielten. Insbesondere Schriften der im letzten Drittel des 18. Jahrhunderts etablierten „Medicinischen Policey“ waren gemäß Lorenz bereits von einem „proto-eugenischen“ Gedankengut geprägt und nahmen dementsprechend Forderungen der Eugenik vorweg – wie etwa die Idee von verpflichtenden Gesundheitszertifikaten für Ehewillige.

Das dritte Kapitel führt aus, wie sich in Frankreich Ideen einer qualitativen Bevölkerungspolitik vor allem in zwei Kontexten herausbildeten. Erstens war dies der Plantagenkolonialismus in Übersee. Um das lukrative Sklavereisystem langfristig zu sichern, schlugen namhafte Staatsbeamte vor, Prinzipien der Tierzucht auf die Kolonien anzuwenden. Sie konzipierten dabei eine koloniale Ständeordnung, die auf einer rassischen Hierarchisierung basierte. Einen zweiten Kontext bildeten die intensiv geführten Bevölkerungsdebatten, die sich während der Revolutionszeit vermehrt Fragen der Qualität zuwandten. Eine wichtige Rolle spielten dabei medizinische Vererbungsdiskurse, die der Gesundheit der Eltern und der Qualität des männlichen Samens bei der Vererbung von Krankheiten ebenso Bedeutung zuschrieben wie der Erziehung, der Ernährung oder Umständen des Beischlafs.

Das kürzere vierte Kapitel ist Großbritannien gewidmet. Dort waren Diskussionen zu *human breeding* wesentlich durch die Verknüpfung von Überbevölkerung und Nahrungsmittelknappheit geprägt, wie sie beim Ökonomen Thomas R. Malthus (1766–1834) besonders einflussreich zum Ausdruck kam. Demgegenüber zeigt das fünfte Kapitel, wie in den USA die Diskurse der Phrenologie als wichtigste Plattform für Ideen zur Reproduktionskontrolle fungierten. So diskutierten Promotoren dieser populären Lehre die Auswirkungen von Rassenmischungen sowie von Verwandtenehen auf die Qualität des Nachwuchses. Wie in Großbritannien kam es indessen auch in den USA zu diskursiven Verknüpfungen von Armut und Erbkrankheiten. In der Folge formulierten Ärzte bereits um die Mitte des 19. Jahrhunderts – also noch vor dem Aufschwung der eugenischen Bewegung – radikale Forderungen, so etwa nach zwangsweisen Kastrationen von Alkoholkranken und Verbrechern.

Das sechste Kapitel schließlich ist einem spezifischen Textgenre gewidmet: den Ehe- und Zeugungsratgebern. Sie zeigen besonders deutlich, wie Ratschläge zur qualitativen Verbesserung des Nachwuchses an Theorien der Vererbung erworbener Eigenschaften gekoppelt waren und an die antike Imaginationstheorie anschließend die besondere Bedeutung eines beidseitig befriedigenden Geschlechtsverkehrs heraushoben. In der Emanzipationsbewegung aktive Ärztinnen folgerten daraus, dass eine Gleichstellung der Geschlechter auch gesunden Nachwuchs sicherstelle. Wie Lorenz argumentiert, bereiteten sich hier bereits im 19. Jahrhundert diskursive Kopplungen zwischen Frauenemanzipation und Eugenik vor.

Lorenz beleuchtet in ihrer gehaltvollen Studie eine enorme Heterogenität von Vererbungs- und Bevölkerungsdiskursen, die von der frühen Aufklärung bis zur Sattelzeit reichen und in denen sich – so die These – bereits proto-eugenische Überlegungen und Projekte herausgebildet hätten. Zwar seien der christliche Gedanke der Caritas sowie die Unklarheit über die Mechanismen der Vererbung für die Umsetzung solcher Ideen hinderlich gewesen. Dennoch hätten die Diskurse zur Menschenzucht mittels „Gewöhnungseffekt“ (319) die Grenzen des Sagbaren immer weiter verschoben, sodass in die Reproduktion eingreifende Maßnahmen zunehmend als erwünscht und somit realisierbar galten. Lorenz zieht dabei eine direkte Kontinuität zur Eugenik: Gegen Ende des 19. Jahrhunderts habe es nur noch entsprechender politischer Rahmenbedingungen und neuer Techniken bedurft, um Ideen der Menschenzucht in die Praxis umsetzen zu können.

Skizziert wird somit ein eher lineares Narrativ, das in einer gewissen Spannung zum präsentierten Material steht. Ideen der Menschenzucht basierten auf sehr unterschiedlichen Vorstellungen von Vererbung und Züchtung, vermengten sich mit heterogenen Reformvorschlägen und gewannen nicht kontinuierlich an Akzeptanz, sondern scheiterten oder versandeten bisweilen auch. Angesichts dieser Vielfalt stellt sich die Frage, ob der nicht weiter geklärte Begriff der „Proto-Eugenik“ als analytische Klammer taugt. Die verschlungenen Diskursverläufe mündeten keineswegs alle und schon gar nicht zwangsläufig in der modernen Eugenik, und im 19. Jahrhundert bildeten sich auch andere, mitunter konkurrierende Begriffe heraus, die je unterschiedliche Vorstellungen von Menschenzucht enthielten. Dazu gehört etwa der Begriff der Puériculture (Schneider 1986) oder auch derjenige der Anthropotechnik.

## Anthropotechnik und Menschenzucht

Diesem Terminus widmet sich die hervorragende Studie *Anthropotechnik: Zur Geschichte eines umstrittenen Begriffs* von Kevin Liggieri. Seine Begriffsgeschichte liest sich besonders gewinnbringend gemeinsam mit der Monografie von Maren Lorenz. Die beiden historischen Untersuchungen zu Visionen der Menschenzucht ergänzen sich nicht nur hervorragend, weil Liggieris Studie zeitlich dort einsetzt, wo Lorenz’ Buch endet, sondern auch, weil sie analytisch zwei unterschiedliche Perspektiven einnehmen. Während Lorenz ein diskurshistorisches Panorama ausbreitet, das begrifflich Heterogenes zusammenführt, folgt Liggieri den schmalen, aber weitverzweigten Spuren eines Begriffs, um die epistemischen Möglichkeitsbedingungen und den Wandel eines züchterischen Zugriffs auf den Menschen zu ergründen.

Begriffshistorisch lässt sich zwischen Anthropotechnik und Eugenik eine Nähe feststellen. So wurden die Termini bisweilen sogar synonym verwendet. Bemerkenswert sind jedoch die Unterschiede: Der Begriff der Anthropotechnik bildete sich nicht nur historisch früher heraus, sein Bedeutungshorizont ging auch oftmals weit über denjenigen der Eugenik hinaus und in einigen Kontexten gewann er gerade in Abgrenzung zur Galton’schen Eugenik an Legitimität. Den Begriff der Anthropotechnik als Knotenpunkt von Diskursen und Bestrebungen zur Humanoptimierung zu untersuchen, ist deshalb ein vielversprechendes Unterfangen. Angesichts seiner Prägung in translingualen und transnationalen Wissensräumen, seinen Adaptionen an unterschiedliche Kontexte sowie seinen vielfältigen Wandlungen stellt er den Begriffshistoriker aber auch vor Herausforderungen. Diese meistert Liggieri methodisch überzeugend, weil er erstens die Begriffsgeschichte konsequent transnational ausrichtet und zweitens Wortgeschichte mit Problemgeschichte verbindet, womit nicht nur semantische Verschiebungen, sondern auch tieferliegende epistemische Veränderungen in den Blick rücken.

Die begriffshistorische Untersuchung nimmt ihren Anfang in Frankreich, wo sich der Terminus *anthropotechnie* im 19. Jahrhundert herausbildete und bereits grundlegende Veränderungen erfuhr (Kapitel 1 bis 3). Anzumerken ist diesbezüglich, dass sich im Französischen sowie auch in weiteren Sprachen – etwa dem Polnischen – eine Assoziation mit der *zootechnie* als Lehre der Tierzucht bereits sprachlich aufdrängt. Wie Liggieri jedoch zeigt, referierte der Begriff zunächst noch nicht auf eine Züchtungslehre im biologischen Sinn, sondern auf eine bildungsphilosophisch orientierte Reflexion. Erst in der zweiten Hälfte des 19. Jahrhunderts kam es zu einer Biologisierung des Begriffs, in deren Zug Anthropotechnik die Bedeutung einer „Tierzucht des Menschengeschlechts“ erhielt. Diesen Wandel führt Liggieri auf die mit Darwin einhergehende Transformation des Sag- und Denkbaren zurück. Erst jetzt sei eine Übertragung von Methoden der Tierzucht auf den Menschen in den Bereich des wissenschaftlich Möglichen und potenziell Machbaren gerückt. Mit Lorenz’ Buch lässt sich dagegen einwenden, dass entsprechende Tierzucht-Analogien bereits vor Darwin verbreitet waren, und zwar auch in medizinischen und naturwissenschaftlichen Texten. Liggieris weiterführende Argumentation ist dennoch plausibel: Er zeigt, wie in Frankreich eine spezifische, lamarckistisch orientierte Darwinrezeption einsetzte, vor deren Hintergrund der Begriff der Anthropotechnik eine neue Prägung erfuhr und an Resonanz gewann, aber auch kontrovers verhandelt wurde. So standen sich um 1900 auf dem „epistemischen Kampfplatz“ (120) der französischen Anthropologie zwei Auffassungen von Anthropotechnik gegenüber. Einerseits erfuhr der Begriff – etwa beim Rassentheoretiker Georges Vacher de Lapouge (1854–1936) – eine erbdeterministische und eugenische Engführung, die auf eine Steuerung der menschlichen Vererbung mittels biotechnischer Eingriffe abzielte. Andererseits setzten andere Autoren – so auch der Anthropologe Léonce Manouvrier (1850–1927) – diesem radikalen Biologismus eine extensive, politisch liberalere Auffassung von Anthropotechnik entgegen, indem sie darunter eine universale Praxis der Menschenverbesserung verstanden, die zwar eugenische Vorhaben inkludierte, aber eine viel umfassendere Verbindung von Medizin, Moral, Erziehung, Recht und Politik anstrebte.

Gerade in dieser zweiten Bedeutungsvariante strahlte der Begriff weit über Frankreich hinaus. Um die Rezeption und unterschiedliche Adaption des Anthropotechnikbegriffs im 20. Jahrhundert zu ergründen, konzentriert sich Liggieri auf drei nationale Kontexte: Polen, Russland und Deutschland (Kapitel 4 bis 6). Eine breite Verwendung fand der Anthropotechnik-Begriff in der polnischen Soziologie und Anthropologie um 1900, wobei hier die biologistisch-rassistischen Züchtungsideen à la Lapouge eine Fortsetzung fanden, der Begriff aber auch Weiterentwicklungen erfuhr. Als besonders einflussreich erwies sich seine Anbindung an marxistische Diskurse. Wie Liggieri überzeugend zeigt, war für sozialistisch orientierte Theoretiker der Begriff der Anthropotechnik deshalb attraktiv, weil er erlaubte, ein Programm der Humanoptimierung zu entwerfen, das eugenische Praktiken einschloss und sich zugleich von der elitär-aristokratischen Galton’schen Eugenik distanzierte. Damit wurde eine wesentliche Grundlage für die Rezeption des Anthropotechnik-Begriffs in Russland geschaffen. Anthropotechnik bildete vor diesem Hintergrund eine Schlüsselkomponente eines sozialistischen Programms der positiven Eugenik, das auf die Schaffung des „neuen Menschen“ ausgerichtet war und sich von der Sterilisationspolitik der westlichen Eugenik abgrenzte. Das Bestreben, unter dem Begriff der Anthropotechnik eine russische Eugenik zu lancieren, fand mit dem Stalinismus jedoch ein abruptes Ende, als solche Vorhaben verboten wurden.

Anders verlief die Rezeption der Anthropotechnik in Deutschland. Zentral war hier insbesondere der Anschluss des Begriffs an die Arbeitswissenschaft. Im Sinne einer rationellen Menschenbeeinflussung und Menschenführung rückte Anthropotechnik dabei in die Nähe des Anfang des 20. Jahrhunderts geprägten Begriffs der Psychotechnik. In Deutschland wurde somit Anthropotechnik weniger in Analogie zur Tierzucht gedacht als mit einer ökonomisch begründeten Menschenoptimierung gleichgesetzt, die auf eine somatische sowie psychisch-geistige Anpassung des Menschen an die Erfordernisse einer hochtechnisierten Arbeitswelt abzielte.

Zu einer eigentlichen „Neuerfindung“ (291) der Anthropotechnik kam es in den 1960er Jahren, wie ein letztes, ebenfalls auf (West‑)Deutschland fokussiertes Kapitel zeigt. Beeinflusst durch Entwicklungen in den USA näherte sich der Begriff weitgehend dem in den 1950er Jahren geprägten Terminus des *human engineering *an*. *Er referierte vermehrt auf eine Gestaltung von Arbeitsplätzen und -geräten mit dem Ziel, diese bestmöglich an die körperlichen und psychologischen Voraussetzungen des Menschen anzupassen. Zwar bezweckte die Anthropotechnik somit weiterhin eine Effizienzsteigerung von Arbeit; statt einer technischen Optimierung des Menschen war nun aber eine Anpassung der Technik an den Menschen das Ziel.

Damit fand geradezu eine Umkehrung der bisherigen Begriffsbedeutung statt. Liggieri zeigt in seiner differenzierten Analyse jedoch immer wieder überzeugend, wie Begriffssemantiken sich nicht einfach ablösten. Vielmehr wirkten ältere Bedeutungsschichten stets weiter, indem sie neuere ergänzten, konkurrierten oder sich mit diesen vermischten. Dies gilt auch für die zweite Jahrhunderthälfte, als sowohl psychotechnische als auch eugenische Praktiken der Humanmodellierung weiterhin zum Bedeutungsfeld der Anthropotechnik gehörten. Liggieris Studie ist eine scharfsinnige Untersuchung, die das beeindruckende Potenzial einer transnational und wissenshistorisch erweiterten Begriffsgeschichte aufzeigt. Aus der Perspektive der Eugenikgeschichte ist die Studie erkenntnisreich, weil sie erhellt, wie sich exklusive, biopolitische Züchtungsvisionen mit inklusiven, universalen Bestrebungen der Humanoptimierung verschränkten. Dies trug zur breiten Anschlussfähigkeit und Akzeptanz von eugenischen Vorhaben bei – auch oder gerade wenn diese unter alternativen, an nationale Diskurse angepassten Begrifflichkeiten und bisweilen in expliziter Distanz zur Eugenik Galtons formuliert wurden.

## Mendelismus und Eugenik

Liggieris und Lorenz’ Studien verdeutlichen, wie Visionen der Menschenzucht auch wesentlich von unterschiedlichen Vererbungsvorstellungen beeinflusst wurden. Erstaunlicherweise taucht jedoch ein bedeutender Protagonist bei Lorenz gar nicht und bei Liggieri nur am Rande auf, wiewohl dieser wie kein anderer sowohl für eine Revolutionierung des Züchtungswesens als auch für eine moderne Konzeption von Vererbung steht: Gregor Mendel (1822–1884). Die sogenannte Wiederentdeckung seiner Vererbungsregeln um 1900 läutete bekanntlich das Zeitalter der modernen Genetik ein. Ihr Einfluss reichte aber weit darüber hinaus. Die Mendelsche Vererbungslehre prägte auch Vorstellungen der menschlichen Vererbung, die Wahrnehmung von Bedrohungen durch Erbkranke und rassisch Fremde sowie die Maßnahmen, mit denen man diese Gefahren unter Kontrolle zu bringen hoffte. Diese politische und gesellschaftliche Wirkmächtigkeit des Mendelismus, sein Einfluss auf die Eugenik und Rassenpolitik ist das Thema von Amir Teichers bedeutendem Buch *Social Mendelism: Genetics and the Politics of Race in Germany, 1900–1948*. Ausgangspunkt seiner Studie ist die Beobachtung einer Vernachlässigung des Mendelismus. Während bisherige historische Narrative die zentrale Rolle des Darwinismus für den Aufstieg der Eugenik betont hätten, sei die Bedeutung des Mendelismus gar nicht oder nur ungenügend erkannt worden. Diese Diagnose ist indes etwas zu pauschal. Gisela Bock vertritt bereits in ihrem Standardwerk aus dem Jahr 1986 die Auffassung, dass der „wichtigste Anknüpfungspunkt“ der deutschen Rassenhygiene nicht etwa Darwin, sondern Mendel gewesen sei (2010: 31). Ähnlich klingt es in Studien zur Eugenik jenseits des Atlantiks: „In the United States, eugenics was informed principally by Mendelianism“ (Stern 2005: 16). Dennoch verweist Teichers Beobachtung auf einen wichtigen Punkt. Während das Weltanschauungspotenzial von Darwins Lehre breit analysiert worden ist – und bisweilen auch zu Verkürzungen wie *From Darwin to Hitler* (Weikart 2004) Anlass gab –, lässt sich zu Mendel nichts Analoges feststellen. Offenbar konnte Mendel den Nimbus des Objektiven und Ideologiefernen bewahren, was auch damit zu tun haben dürfte, dass seine Lehre weniger als theoretische Abhandlung angesehen wurde denn als gleichsam mathematisch formalisierte Gesetze. Hier setzt Teichers Studie an: Gerade indem der Mendelismus einen simplen Mechanismus für die Übertragung von Erbfaktoren postulierte, erwies er sich als anschlussfähig an politische Ideologien und eugenische Projekte. Innovativ ist seine Studie auch deshalb, weil er mit dem in Analogie zum Sozialdarwinismus gebildeten Begriff des *social mendelism* einen analytischen Rahmen schafft, um solche Übertragungen von Mendels Lehre auf Mensch und Gesellschaft zu untersuchen.

Die beiden ersten Kapitel gehen der Frage nach, wie mendelistische Konzepte in humanwissenschaftliche Forschungsfelder Eingang fanden und dabei im frühen 20. Jahrhundert eine neue Ära der menschlichen Erbforschung einläuteten. Konkret behandelt Teicher die Aufnahme des Mendelismus in drei Disziplinen – nämlich die Genealogie, die Psychiatrie und die Anthropologie. Erstere tat sich schwer, den Mendelismus zu integrieren – unter anderem, weil sein Fokus auf wenige Generationen einen der wesentlichen Antriebe der Genealogie, nämlich die Suche nach weit zurückliegenden Verwandten, zu unterminieren drohte. Demgegenüber übte Mendels Lehre einen prägenden Einfluss auf Psychiatrie und Anthropologie aus, und zwar auch deshalb, weil sie – so Teichers plausible These – zu zentralen Fragen der beiden Disziplinen beitrug. Im ersten Fall war dies die Klassifizierung von psychiatrischen Krankheiten, im zweiten ein neuer Zugang zu Fragen der menschlichen Rassen, die nun weniger als holistische Entitäten denn als Kombination von unabhängig vererbten Körpermerkmalen verstanden wurden. Die beiden ersten Kapitel rekonstruieren die Anfänge einer mendelistischen Humangenetik konzis, behandeln aber auch bereits Bekanntes – so Eugen Fischers (1874–1967) Bastardstudien, Ernst Rüdins (1874–1952) Erbforschungen oder Ottokar Lorenz’ (1832–1904) wissenschaftliche Genealogie. Danach verlässt Teicher diese vorgespurten Pfade, indem er dem Mendelismus in breiteren Kontexten von Politik und Gesellschaft nachspürt. Nun zeigt sich, wie produktiv und erkenntnisreich sein Unterfangen ist, die Geschichte von Eugenik und Rassenpolitik unter dem Gesichtspunkt des *social mendelism* neu zu beleuchten. Das dritte Kapitel behandelt die Mobilisierung des Mendelismus für die Rassenhygiene und das faschistische Projekt einer nationalen Wiedergeburt. Zum einen war es die Idee der Reinheit, die den Mendelismus für diese Ideologien anschlussfähig machte. So konnte Rassenreinheit unter Verweis auf wissenschaftliche Autorität neu definiert werden als Homozygotie beziehungsweise Reinerbigkeit. Die unterschiedlichen Bezugnahmen auf Mendel in den Diskussionen um Rassenmischungen zeugen jedoch auch von der politischen Polyvalenz des *social mendelism*. Zum anderen ließ sich das Konzept der Rezessivität mit medizinischen und sozialen Ängsten aufladen. Rezessiv vererbte Erbkrankheiten galten als besonders gefährlich, da rezessive Gene im Verborgenen blieben und schwieriger zu kontrollieren seien. Des Weiteren arbeitet Teicher überzeugend heraus, wie die Vorstellung von rezessiven Genen geeignet war, antisemitische Bedrohungsängste zu aktivieren und zu verstärken. So korrespondierte die Identifikation von Juden mit Rezessivität mit antisemitischen Stereotypen, die Juden eine nur scheinbare Assimilation und ein verborgenes Spiel der Täuschung unterstellten.

Kapitel 4 und 5 behandeln die Zeit des Nationalsozialismus. Dabei analysiert Teicher erstens, welche Bedeutung dem Mendelismus bei der Implementierung und Umsetzung eugenischer und rassistischer Gesetze zukam. So arbeitet er heraus, wie der Mendelismus nicht nur die Ausrichtung der Sterilisationskampagne prägte, sondern auch eine wesentliche Grundlage für die Nürnberger Rassengesetze bildete. Gemäß Teicher spielten mendelistische Konzepte geradezu eine Schlüsselrolle dabei, wie Nationalsozialisten die Grenzen zwischen Juden und Nichtjuden zogen und begründeten. Zweitens behandelt er den Mendelismus als Unterrichtsthema in Schulen. Besonders eindrücklich sind dabei die Ausführungen, die auf Berichten von angehenden Lehrern basieren. Sie zeigen einerseits, wie die Referenz auf Mendel auch nützlich war, um eine ideologisch gewollte Einheit im Klassenzimmer herzustellen. So ließ sich mit Verweis auf Mendels Gesetze erklären, warum einige nicht-nordisch aussehende Schüler trotzdem „nordisches Blut“ aufweisen würden. Der mendelistisch untermauerte Eugenikunterricht führte aber auch zu ungewollten Reaktionen. So berichteten Lehrer etwa von einer „Vererbungsfurcht“ mancher Schüler, die in der Folge keine Informationen über ihre Familiengeschichte preisgeben wollten. Im Prolog öffnet Teicher die Perspektive und diskutiert die Frage, inwiefern es sich beim *social mendelism* um ein trans- beziehungsweise internationales Phänomen handelte, wobei er diesbezüglich insbesondere Forschungsdesiderate feststellt.

Teichers grundlegende Studie zeigt, wie der Mendelismus eine wesentliche Rolle beim Aufstieg, der Ausprägung und der Radikalisierung der deutschen Rassenhygiene spielte. Was in Bezug auf den Darwinismus längst bekannt ist, arbeitet Teicher differenziert argumentierend für den Mendelismus heraus: Er stellte ein Reservoir von Bildern, Metaphern und Argumenten bereit, aus denen Nationalsozialisten schöpften, um ihre Rassen- und Sterilisationspolitik wissenschaftlich zu legitimieren. Teichers Narrativ folgt dabei keinem gradlinigen Weg; keinem „von Mendel zu Hitler“. Vielmehr betont er immer wieder die Widersprüche, Polyvalenzen und Kontingenzen, die den politischen Gebrauch des Mendelismus bestimmten. Gerade dadurch schärft Teichers Studie unseren Blick für die ambivalenten und historisch sich wandelnden Verschränkungen von Wissenschaft und Ideologie.

Die hier vorgestellten Studien vermögen den Blick auf die Geschichte der Eugenik und der Menschenzucht in mehrerlei Hinsicht zu erweitern. Erstens öffnen sie die Perspektive auf historisch weiter zurückreichende Entwicklungslinien. Als wissenschaftliche und ideologische Strömung ist die Eugenik zwar erst Ende des 19. Jahrhunderts entstanden, ihre Ideen und vorgeschlagenen Maßnahmen haben aber bisweilen eine lange und verschlungene Vorgeschichte: Insbesondere in der Sattelzeit bildeten sich bereits Konzepte der Vererbung, der Humanoptimierung und der Fortpflanzungskontrolle heraus, die für eine Genealogie von Eugenik und Menschenzucht im 20. Jahrhundert wesentlich sind. Für eine solche Genealogie erweist es sich zweitens als produktiv, historische Zusammenhänge jenseits des üblichen Fokus auf Darwin und Galton herauszuarbeiten. Die Studie zum Mendelismus erhellt wenig bekannte Verbindungen von Eugenik, Rassenanthropologie und Antisemitismus und die Begriffsgeschichte der Anthropotechnik bringt nicht zuletzt neue transnationale Beziehungen zum Vorschein: Nicht Großbritannien und Deutschland stehen am Anfang der hier beleuchteten Menschenzuchtsvisionen, sondern Frankreich, Polen und Russland. Schließlich bieten die besprochenen Studien einen produktiven Anschluss an die Wissensgeschichte, indem sie die Wissenschaftsgeschichte insofern erweitern, als sie die Produktion und Zirkulation von Vererbungs- und Züchtungswissen jenseits von universitärer Forschung in den Blick nehmen. Sie spüren wissenschaftlichen Begriffen und Ideen in breiteren Wissenskontexten nach, folgen ihnen in literarischen Schriften, Rechtstexten und Ratgebern oder rekonstruieren ihren Gebrauch in Schulzimmern, Gerichten und staatlichen Bürokratien. Gerade aufgrund solcher wissenshistorischer Perspektiven bieten die Studien wertvolle, neue und bisweilen überraschende Einsichten in die heterogene Entstehung, Legitimierung und Radikalisierung von Programmen der Eugenik und der Menschenzucht.
